# Invasive and echocardiographic gradients of self- and balloon-expandable valves in failing aortic bioprostheses

**DOI:** 10.1007/s12471-026-02061-7

**Published:** 2026-08-18

**Authors:** Sraman Chatterjee, Mark van den Dorpel, Rik Adrichem, Claire Ben Ren, Isabella Kardys, Rutger-Jan Nuis, Joost Daemen, Alexander Hirsch, Marcel L. Geleijnse, Nicolas M. Van Mieghem

**Affiliations:** 1 Department of Cardiology, Cardiovascular Institute, Erasmus University Medical Center, Rotterdam, The Netherlands; 2 Department of Radiology and Nuclear Medicine, Erasmus University Medical Center, Rotterdam, The Netherlands; 3 Interventional Cardiology, Department of Cardiology, Thoraxcenter, Erasmus University Medical Center, office Nt-645 Rotterdam, The Netherlands

**Keywords:** TAVI, Echocardiography, Hemodynamics

## Abstract

**Background:**

Transcatheter aortic valve implantation (TAVI) is an established treatment for a failing aortic bioprosthesis. The hemodynamics of TAVI in degenerated transcatheter or surgical aortic valves (TAV-in-TAV or TAV-in-SAV) are unknown. We aimed to investigate hemodynamic differences between self- and balloon-expandable TAV-in-TAV and TAV-in-SAV groups, and transthoracic echocardiography-derived (TTE) versus invasive transaortic pressure gradients.

**Methods:**

Patients ≥ 18 years with a self-expanding EVOLUT (SEV) or balloon-expandable SAPIEN3 (BEV) valve for TAV-in-TAV or TAV-in-SAV were included. Transaortic gradients were determined invasively and by TTE within 48 h post-intervention.

**Results:**

We identified 56 patients with SEV (*n* = 36) or BEV (*n* = 20) TAV in a failing aortic bioprosthesis. Fourteen cases involved failing transcatheter valves and 42 surgical bioprostheses. Invasive mean gradients were similar after BEV and SEV (median (25th–75th percentile): 6.0 (1.5–6.5) vs 7.0 (2.0–10.0) mm Hg, *p* = 0.109). Mean gradients by TTE were higher for BEV than SEV at discharge (16.0 (10.8–19.8) versus 10.0 (7.0–12.0) mm Hg, *p* = 0.003) and 12-month follow-up (13.0 (11.0–15.5) versus 9.0 (7.0–12.3) mm Hg, *p* = 0.025). The discrepancy between TTE and invasive mean gradients was significantly larger for BEV than SEV (13.0 (6.3–14.0) vs 3.0 (1.5–8.3) mm Hg, *p* = 0.001) and most pronounced in TAV-in-SAV (BEV 13.0 (6.3–17.0) vs SEV 3.0 (1.0–8.5) mm Hg, *p* = 0.004).

**Conclusion:**

Mean gradient after TAV in a failing bioprosthesis is similar for BEV and SEV, when measured invasively, but higher with BEV than SEV by TTE. The discordance between invasive and TTE-derived mean gradients is the largest in BEV.

**Supplementary Information:**

The online version of this article (10.1007/s12471-026-02061-7) contains supplementary material, which is available to authorized users.

## Introduction

Transcatheter aortic valve implantation (TAVI) is an established therapy for elderly patients with symptomatic severe aortic stenosis (AS) [1–8]. Bioprosthetic transcatheter and surgical valve failure may occur over time [9–11]. Surgery as treatment option for a failing surgical or transcatheter bioprosthesis carries a higher risk than redo-TAVI [12, 13].

The self-expanding supra-annular EVOLUT (Medtronic plc, Minneapolis, Minnesota) valve (SEV) and balloon expandable intra-annular SAPIEN3 (Edwards Lifesciences, Irvine, California) valve (BEV) are used as transcatheter treatment options in a failing surgical aortic valve (TAV-in-SAV) or a failing transcatheter aortic valve (TAV-in-TAV) [8, 14–18]. In patients with native aortic valve disease, SEV TAVI is associated with better and BEV TAVI with similar hemodynamic valve performance, compared to surgery [19, 20].

The residual transaortic gradient is typically higher for TAV in a failing bioprosthesis than for TAV in a native aortic valve [21, 22]. Little is known about the hemodynamic consequences of SEV versus BEV in the context of TAV in a failing surgical or transcatheter valve, and whether there is a difference between invasive and TTE assessment [22].

Therefore, our aim was to investigate the difference in hemodynamic performance between SEV and BEV in patients undergoing TAV-in-TAV and TAV-in-SAV and evaluate the difference between transaortic gradients measured invasively and by TTE.

## Methods

### Ethical statement

All patients consented to the use of information (including imaging and procedural data) for research purposes. All data were obtained from electronic patient records and collected in a dedicated institutional database. The study was approved by the institutional review board (METC, ID number: MEC-2023-0419) in September 2023.

### Study design and population

We performed a single-center retrospective study. We included all patients who underwent TAV-in-TAV or TAV-in-SAV with the EVOLUT SEV or SAPIEN3 BEV in the Erasmus University Medical Center between 2014 and 2022. The treatment strategy and transcatheter valve selection were determined by the multidisciplinary heart team. TAVI procedures were performed under local anesthesia to local clinical practice. Transaortic gradients were determined invasively before and after the actual transcatheter valve implantation using 2 6F fluid-filled pigtail catheters; one positioned at the level of the mid ventricle and in the aorta at 2 cm above the balloon expandable frame or at the level of the upper quadrant of self-expanding valves. TTE was performed within 48 h post-TAVI, at 30 days, and 12 months.

### Statistical analysis

Distributions of continuous variables were tested for normality by using the Shapiro-Wilk test. Continuous variables are expressed as median (25th–75th percentile) or mean±SD, depending on normal distribution. Categorical variables are expressed as numbers and percentages. Comparison of continuous variables between groups was performed using Student’s *t*-test or the Mann-Whitney *U* test, depending on data distribution. Comparisons of categorical variables between groups were performed with chi-square tests or Fisher’s exact tests, as appropriate. Additional subgroup analyses were performed, comparing the subgroups (TAV-in-TAV and TAV-in-SAV) with respect to echocardiographic and invasive gradients, and the discordance between these modalities was determined using the aforementioned statistical techniques. The gradient discordance between different valve sizes (very small (XS), small (S), medium (M), and large (L)) was also compared. Two-sided P values < 0.05 were considered statistically significant. Analyses were performed using SPSS (28.0, Armonk, YU, USA; IBM Corp.).

## Results

### Baseline characteristics

**Fig. 1:**
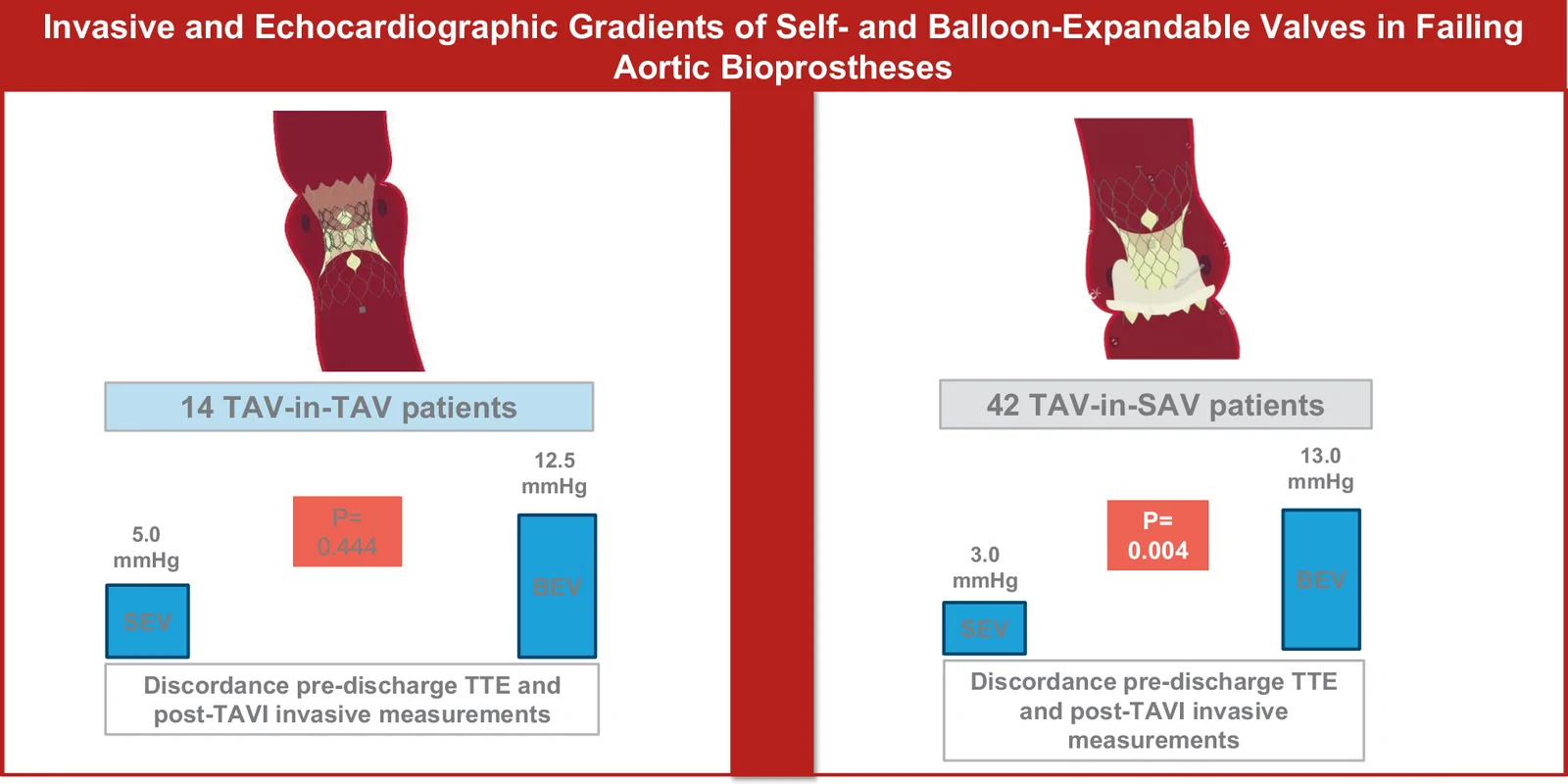
Infographic

**Fig. 2:**
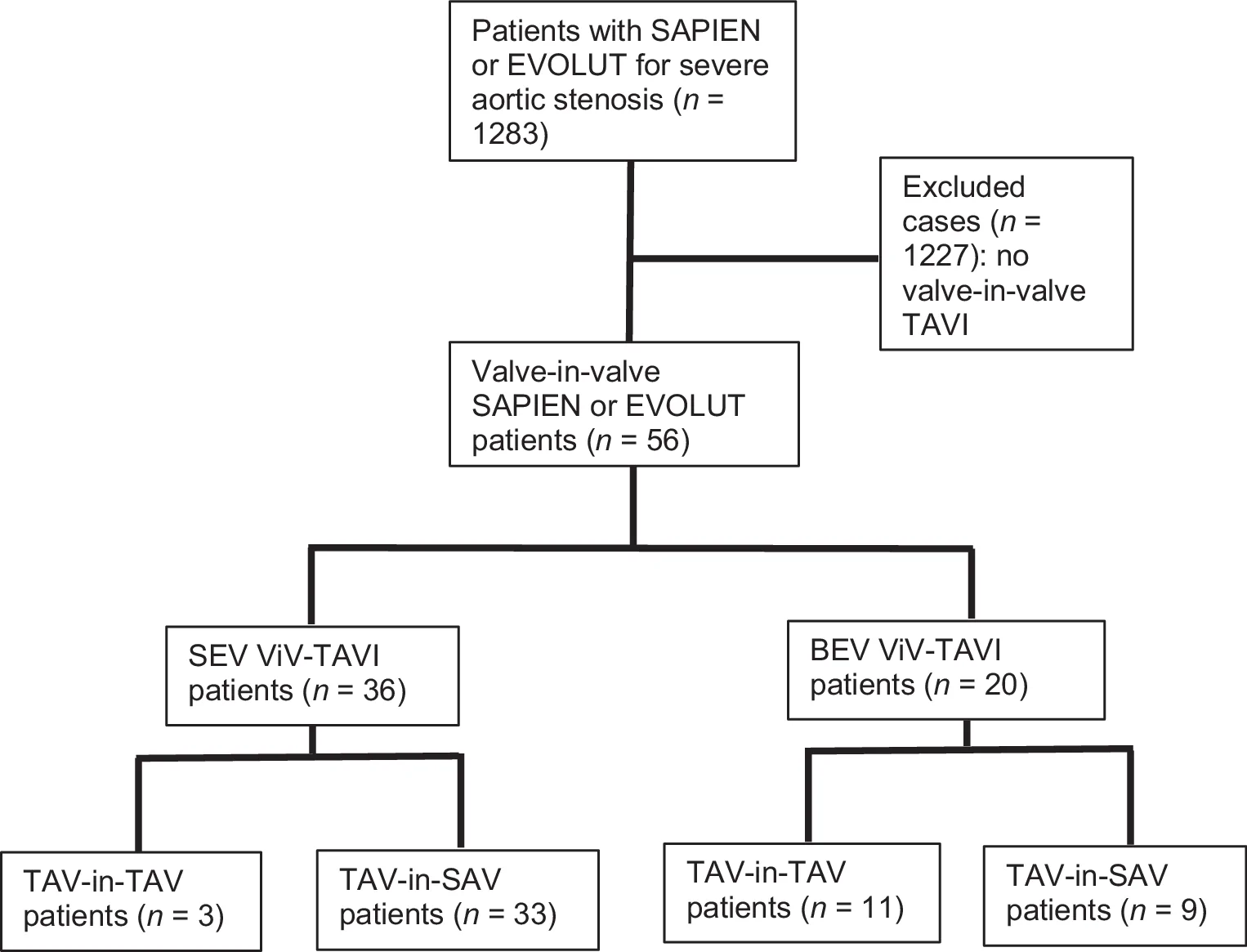
Patient selection flowchart

We identified 1283 patients with a SAPIEN3 or EVOLUT valve, of which 56 patients were extracted with a self-expanding (*n* = 36) or balloon-expanding (*n* = 20) valve in a failing bioprosthesis (Tab. 1). Fourteen cases involved a failing transcatheter valve and 42 a failing surgical bioprosthesis (Figs. 1 and 2).

**Table 1:** Baseline characteristics

	SEV (*n* = 36)	BEV (*n* = 20)	*P* value
Age (y)	79.0 (76.0–84.0)	75.0 (67.0–76.8)	**0.002**
Male	27 (75%)	11 (55%)	0.125
BMI (kg/m^2^)	24.8 (22.5–27.5)	23.8 (22.8–25.4)	0.389
BSA (m^2^)	1.9 (1.8–2.0)	1.7 (1.6–1.7)	**0.001**
STS	4.6 (2.9–6.8)	4.7 (2.5–6.2)	0.596
Hypertension	28 (78%)	12 (60%)	0.285
Hypercholesterolemia	23 (64%)	13 (65%)	0.430
Atrial fibrillation	12 (33%)	4 (20%)	0.365
Diabetes	6 (17%)	6 (30%)	0.182
Smoking	23 (64%)	8 (40%)	**0.047**
Renal failure	14 (39%)	9 (45%)	0.487
*Antithrombotic regiment*^*a*^			
Aspirin	12/33 (36%)	8/17 (47%)	0.470
Clopidogrel	9/33 (27%)	3/17 (18%)	0.456
VKA	6/33 (18%)	3/17 (18%)	0.965
NOAC	6/33 (18%)	3/17 (18%)	0.965
*NYHA Class*^*a*^			
NYHA 1	1/32 (3%)	1/16 (6%)	0.610
NYHA 2	6/32 (19%)	2/16 (13%)	0.584
NYHA 3	19/32 (59%)	10/16 (63%)	0.835
NYHA 4	6/32 (19%)	3/16 (19%)	1.000
*Echo characteristics*			
LV EF (%)	51.5 (40.0–60.0)	45.0 (35.8–56.0)	0.167
LVEF > 50%^a^	16/30 (53%)	4/13 (31%)	0.173
LVEF 40–50%^a^	8/30 (27%)	5/13 (38%)	0.439
LVEF < 40%^a^	6/30 (20%)	4/13 (31%)	0.443
Mean aortic gradient > 20 mm Hg	17 (47%)	8 (40%)	0.931
AVA (cm^2^)	1.5 (1.0–1.6)	1.0 (0.7–1.7)	0.187
SVI (ml/m^2^)	46.9 (38.8–58.6)	52.7 (44.1–66.5)	0.801
*Aortic regurgitation*^*a*^			
None	3/31 (10%)	2/16 (13%)	Overall *p*‑value ***=*** 0.359
Trace	4/31 (13%)	3/16 (19%)	
Mild	1/31 (3%)	4/16 (25%)	
Moderate	17/31 (55%)	5/16 (31%)	
Severe	6/31 (19%)	2/16 (13%)	
*Indication for TAVI*			
Surgical failed bioprosthesis	33 (92%)	9 (45%)	**0.001**
Degenerated TAVI bioprosthesis	3 (8%)	11 (55%)	**0.001**
*Valve types*			
Evolut R	19 (53%)	0 (0%)	**0.001**
Evolut Pro(+)	17 (47%)	0 (0%)	**0.001**
Edwards Sapien 3	0 (0%)	20 (100%)	**0.001**
*Valve size group*			
Very small (XS) (Sapien3 20 mm or Evolut 23 mm)	4 (11%)	1 (5%)	Overall *p*‑value **=** **0.049**
Small (S) (Sapien3 23 mm or Evolut 26 mm)	17 (47%)	9 (45%)	
Medium (M) (Sapien3 26 mm or Evolut 29 mm)	15 (42%)	6 (30%)	
Large (L) (Sapien3 29 mm or Evolut 31/34 mm)	0 (0%)	4 (20%)	

Values are median (25th–75th percentile) or *n* (%)

*BEV* balloon-expandable valve, *BMI* body mass index, *SEV* self-expanding valve, *BSA* body surface area, *STS* Society of Thoracic Surgeons predicted risk of mortality, *EF* ejection fraction (%), *AVA* aortic valve area (cm^2^), *SVI* stroke volume index (ml/m^2^)

^a^ Contains missing data due to incomplete patient records

Most patients with a failing surgical bioprosthesis were treated with SEV. Conversely, most patients with a failing transcatheter valve received a BEV. The majority of patients with a failed SEV received a BEV (89%), and patients with a degenerated BEV were most frequently treated with a SEV (67%). SEV patients were significantly older than BEV patients (79 vs. 75 years, *p* = 0.002). Smaller valves were more often treated with SEV than BEV.

### Hemodynamics

**Fig. 3:**
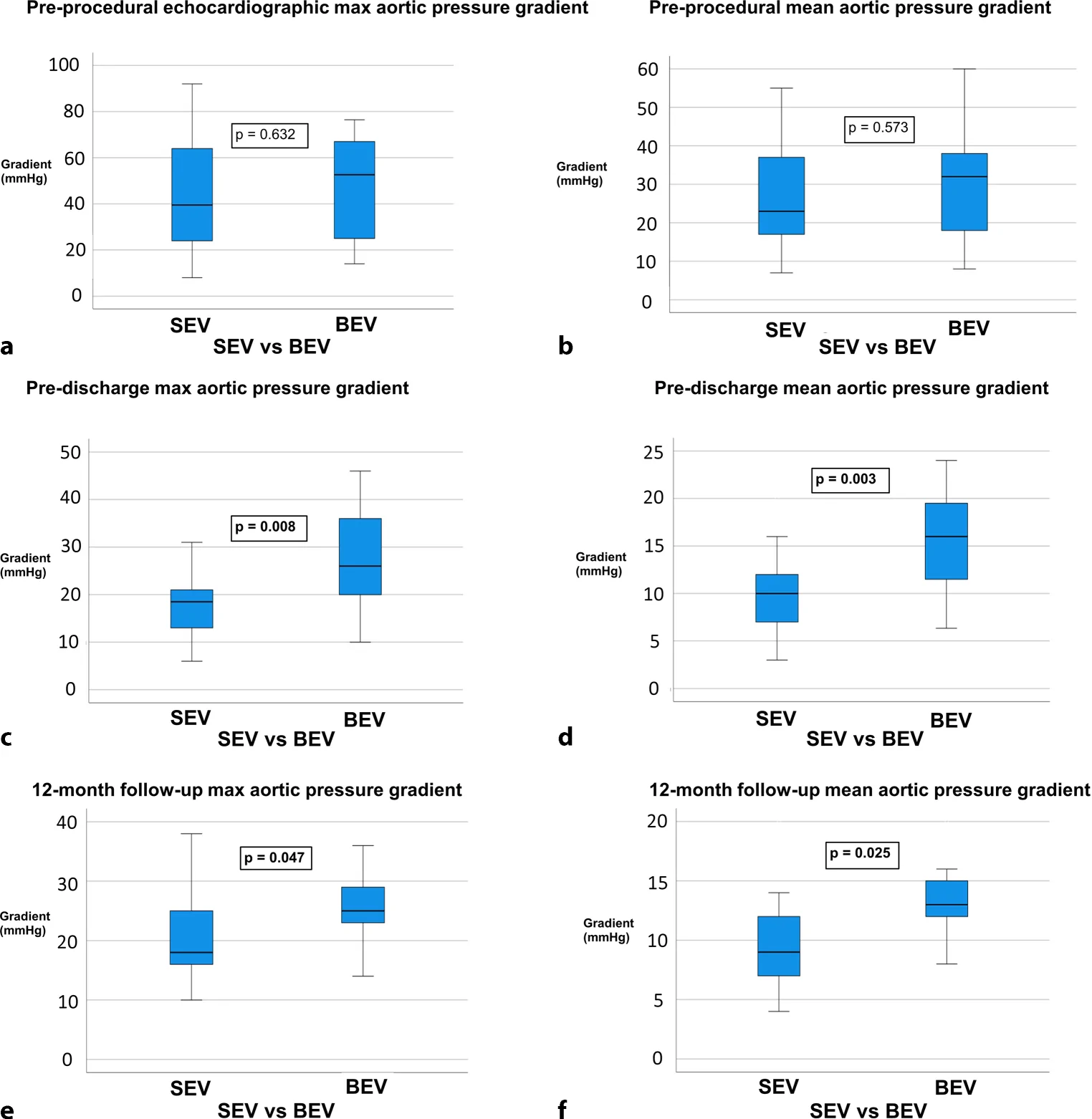
Pressure gradients measured by transthoracic echocardiography (TTE) in self-expanding (SEV) vs balloon-expanding (BEV) valve-in-valve (ViV) patients

**Fig. 4:**
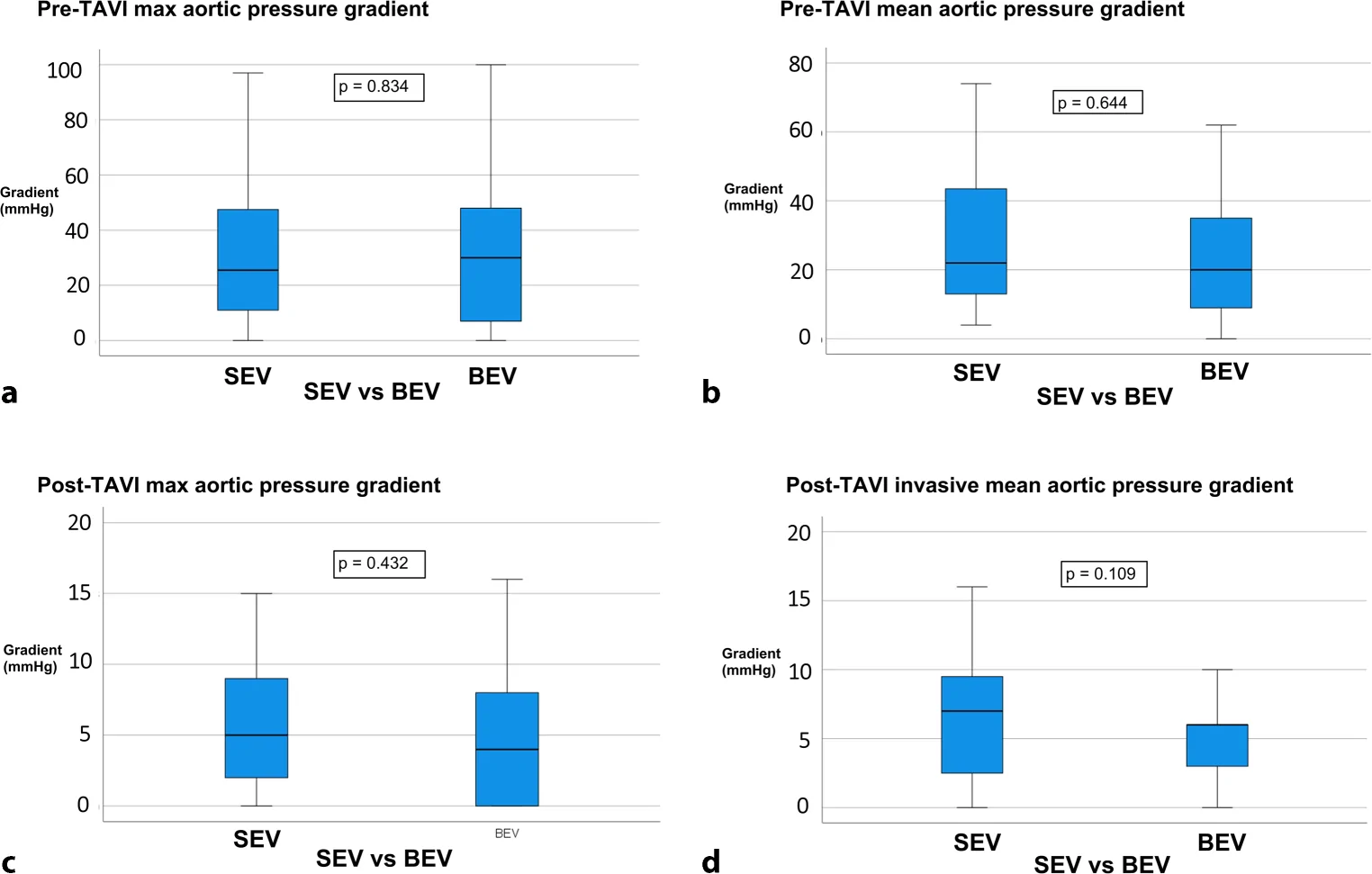
Pressure gradients measured invasively in self-expanding (SEV) vs balloon-expanding (BEV) valve-in-valve (ViV) patients

Transaortic gradients before and after the TAV-in-SAV or TAV-in-TAV procedure are tabulated in Tab. 2 and Figs. 3 and 4. Pre- and post-TAVI invasive mean gradients were similar for BEV and SEV. BEV patients had significantly higher mean gradients by pre-discharge echocardiography than SEV patients (16.0 (10.8–19.8) versus 10.0 (7.0–12.0) mm Hg, *p* = 0.003).

**Table 2:** Pre- and post-procedural invasive and echocardiographic measurements in transaortic pressure gradient

	SEV (*n* = 36)	BEV (*n* = 20)	*P* value
*Invasive measurements*			
*Pre-TAVI*			
Mean aortic gradient (mm Hg)	22.0 (13.0–45.3)	20.0 (8.5–36.5)	0.644
Peak aortic gradient (mm Hg)	25.5 (9.5–49.3)	30.0 (4.0–50.5)	0.834
*Post-TAVI*			
Mean aortic gradient (mm Hg)	7.0 (2.0–10.0)	6.0 (1.5–6.5)	0.109
Peak aortic gradient (mm Hg)	5.0 (2.0–9.0)	4.0 (0.0–9.0)	0.432
*Echocardiographic measurements*			
*Pre-TAVI*			
Mean aortic gradient (mm Hg)	23.0 (16.0–37.0)	32.0 (15.5–42.2)	0.588
Peak aortic gradient (mm Hg)	39.5 (23.2–65.0)	52.6 (25.0–67.6)	0.632
*Pre-discharge*			
Mean aortic gradient (mm Hg)	10.0 (7.0–12.0)	16.0 (10.8–19.8)	*0.003*
Peak aortic gradient (mm Hg)	18.5 (13.0–21.0)	26.0 (19.5–37.0)	*0.008*
*12 months*^*a*^			
Mean aortic gradient (mm Hg)	9.0 (7.0–12.3)	13.0 (11.0–15.5)	*0.025*
Peak aortic gradient (mm Hg)	18.0 (15.5–25.0)	25.0 (15.5–29.0)	*0.047*
*Difference between gradients*			
Pre-discharge echo mean gradient—post-procedural invasive mean gradient (mm Hg)	3.0 (1.5–8.3)	13.0 (6.3–14.0)	*0.001*
12 months echo mean gradient—pre-discharge echo mean gradient (mm Hg)	0.0 (−3.5–3.0)	−1.0 (−5.5–3.0)	0.594

Values are median (25th–75th percentile). *BEV* balloon-expandable valve; *SEV* self-expanding valve;

^a^Gradient data available of *n* = 26 SEV and *n* = 13 BEV at 12 months (otherwise lost to follow-up/death)

A similar trend was visible at 12-month follow-up (13.0 (11.0–15.5) versus 9.0 (7.0–12.3) mm Hg, *p* = 0.025). The discrepancy in mean gradient by pre-discharge TTE versus invasive measurement was significantly larger with BEV than SEV (13.0 (6.3–14.0) vs. 3.0 (1.5–8.3) mm Hg, *p* = 0.001).

This discrepancy in mean gradient by pre-discharge TTE versus invasive measurement was most pronounced with BEV TAV-in-SAV compared to SEV (13.0 (6.3–17.0) vs. 3.0 (1.0–8.5), *p* = 0.004). A similar trend was visible in the TAV-in-TAV group (BEV: 12.5 (6.3–13.8) vs. SEV: 5.0 (5.0–5.0), *p* = 0.444) (Tab. 3).

**Table 3:** Degenerated bioprosthesis cohort and pre- and post-procedural invasive and echocardiographic measurements in transaortic pressure gradient

Degenerated valve	SEV + BEV	*P* value^a^	SEV	*P* value^a^	BEV	*P* value^a^	*P* value (SEV vs. BEV)
*Post-procedural invasive mean gradient*							
TAV-in-TAV	6.0 (1.8–8.0)	0.510	4.0 (1.0–4.0)	0.422	6.0 (2.0–8.0)	0.277	0.864
TAV-in-SAV	6.0 (2.3–9.0)		7.5 (2.3–10.0)		4.5 (0.8–6.0)		0.059
*Pre-discharge TTE mean aortic gradient*							
TAV-in-TAV	13.0 (11.0–19.5)	0.062	9.0 (9.0–9.0)	0.828	14.5 (13.0–20.3)	1.000	0.444
TAV-in-SAV	10.0 (7.3–15.0)		10.0 (7.0–12.0)		17.0 (9.3–19.8)		0.083
*Discordance between pre-discharge TTE mean gradient and post-procedural invasive mean gradient*							
TAV-in-TAV	12.0 (5.0–13.5)	0.065	5.0 (5.0–5.0)	0.692	12.5 (6.3–13.8)	0.536	0.444
TAV-in-SAV	3.0 (2.0–10.0)		3.0 (1.0–8.5)		13.0 (6.3–17.0)		*0.004*

^a^For comparisons between TAV-in-TAV and TAV-in-SAV.

Values are median (25th–75th percentile). *BEV* balloon-expandable valve; *SEV* self-expanding valve

Subgroup analyses comparing echocardiography-invasive measurement discordance for different valve size categories displayed a larger discrepancy in smaller valve sizes in SEV: Very small (XS) = 14.0 (5.0–NA); Small (S) = 2.0 (−2.0–6.5) mm Hg, *p* = 0.025, and Medium (M) = 3.0 (1.3–6.5) mm Hg, *p* = 0.049. There were no differences for the various BEV valve sizes (Electronic Supplementary Material [ESM] Table S1).

The comparison between SEV and BEV for each valve size category yielded a significantly larger modality discordance for the BEV group, only within the Small valve subgroup: BEV = 13.5 (10.8–16.3) vs. SEV = 2.0 (−2.0–6.5), *p* < 0.001 (ESM Table S1).

Exploratory analyses into the evolution of echocardiography-invasive discordance measures over time in both SEV and BEV illustrated no significant differences between procedure dates.

### Clinical outcomes

At 30-day and 12-month follow-up, clinical outcome data were available for 45 patients (31 SEV and 14 BEV) and 43 patients (30 SEV and 13 BEV), respectively. There were no differences in clinical outcomes at 30 days and 12 months between the SEV and BEV groups (ESM Table S2).

## Discussion

Main findings of our study that compared TAV with BEV vs. SEV in failing bioprostheses are: 1) There was no difference between BEV and SEV in terms of invasively measured mean gradients. 2) Mean gradients by pre-discharge and 12-month TTE were significantly higher for BEV than SEV. 3) The discrepancy between pre-discharge echocardiography and invasively measured mean gradients was larger for BEV than SEV. 4) The discrepancy between TTE and invasive measurements was more pronounced for BEV TAV-in-SAV compared to SEV.

Several studies have explored transvalvular gradient differences between SEV and BEV and reported findings that are consistent with our results. Van den Dorpel et al. found similar mean gradients by invasive assessment but higher mean gradients by pre-discharge and 12-month TTE with BEV as compared to SEV in native anatomies [23]. Also, the discrepancy between TTE-derived and invasive gradients post-TAVI was more pronounced with BEV than SEV and accentuated in the smallest anatomies.

For the first time, we examined echocardiography-invasive gradient discordance for SEV and BEV platforms simultaneously for both TAV-in-SAV and TAV-in-TAV patients. The LYTEN trial compared hemodynamic performance with SEV and BEV in failing small (≤ 23 mm) surgical aortic bioprostheses [24] and concluded that SEV TAV-in-SAV resulted in lower mean gradients and superior hemodynamic performance (mean gradient < 20 mm Hg, peak velocity < 3 m/s, Doppler velocity index ≥ 0.25, and less than moderate AR) by TTE at 1‑year follow-up. They also explored differences in invasive gradients between SEV and BEV and found no significant differences in mean or peak invasive gradients, which is consistent with our findings.

Other reports also documented this discordance between gradient measurement modalities (echocardiography versus invasive), with consistently higher gradients by TTE in both SEV and BEV [25, 26]. The discordance occurred with TAV-in-native aortic stenosis and TAV-in-SAV. Abbas et al. also concluded that the discordance between invasive and echocardiographic measurements was visible mainly after TAVI in failed surgical bioprostheses [26].

Each measurement modality has inherent pitfalls and limitations. For the invasive assessment, optimal catheter positioning in the LV and ascending aorta is still debated. Sidehole catheters may not capture the maximal gradient and hemodynamic changes due to filling status, transient cardiac stunning post-TAVI, and the use of sedatives/anesthetics may affect flow and ad hoc gradients [25, 27]. Barker et al. proposed a standardized and reproducible protocol for invasive assessment, which may eliminate carriable effects of pressure recovery [28].

TTE assessment can be hampered by improper alignment of the ultrasound beam, and there is no clear consensus concerning the positioning of the Doppler beam with different THV platforms. Furthermore, echocardiography relies on the simplified Bernoulli’s principle that discards flow acceleration, turbulence, and viscous friction components [29, 30]. Unlike invasive assessment, TTE measures the mean gradient at the level of the vena contracta and therefore, does not include pressure recovery in the ascending aorta [31–36]. Collectively, this may explain inaccuracies and different echo Doppler mean gradient recordings with SEV and BEV [31, 37–40].

Fundamental differences in transcatheter valve design may explain the more pronounced discordance between measurement modalities with BEV than SEV. SEV features a longer metal frame that flares open into the ascending aorta and has supra-annular implanted valve leaflets. Conceivably, this configuration may increase turbulence around the vena contracta and reduce pressure recovery in the aorta. Consequently, there may be smaller differences between invasive and TTE-derived gradients with SEV than the more tubular and shorter BEV design [40].

Our observation of larger discordance between invasive and TTE gradients with smaller transcatheter valves is consistent with previous work, which found that small valve sizes (Medtronic self-expanding valve (SEV) ≤ 26 mm or Edwards balloon expanding (BEV) ≤ 23 mm) were independently associated with elevated transvalvular mean gradients and an absolute discordance between these modalities of > 10 mm Hg [26].

The Redo-TAVI registry investigated outcomes of self-expanding and balloon-expandable TAV-in-TAV [14]. Patients with BEV TAV-in-TAV had a smaller effective orifice area and correspondingly higher residual mean gradients on TTE compared with SEV. This resonates with our findings as BEV patients had higher residual gradients than SEV patients, particularly in smaller anatomies.

### Clinical implications

The observation of higher echocardiographic gradients in BEV-treated patients, despite similar invasive hemodynamics, may have clinical relevance, particularly in patients with small annuli or failed bioprostheses. Higher residual gradients, smaller valve prosthesis, and valve-in-valve procedures are associated with an increased risk of patient–prosthesis mismatch and may contribute to higher mortality rates and HF rehospitalization, although definitive evidence in the TAV-in-SAV and TAV-in-TAV settings is still lacking [41–43].

However, a recent study indicated that only invasive gradients, not echocardiography-derived gradients, correlated with all-cause mortality at 30 days, 1 year, and 2 years [23]. This reinforces the clinical significance of using invasive measurements for accurate prognostication, especially in valve-in-valve procedures, where gradient mismatch is a concern.

In our cohort, the sample size was too small to draw definite conclusions regarding clinical outcomes between SEV and BEV in the context of valve-in-valve procedures. Given the growing numbers of reinterventions and valve-in-valve procedures, careful attention to post-procedural gradients and valve selection is warranted. Future prospective studies with long-term follow-up are needed to clarify whether invasive and echocardiographic gradients after valve-in-valve procedures correlate with valve durability and clinical outcomes, and to better define acceptable thresholds for residual gradients in this setting.

### Limitations

This retrospective single-center study has inherent limitations. The study included failing surgical and transcatheter valves. The number of cases was too small to unravel potential differences between TAV in surgical and transcatheter valves, respectively. Furthermore, SEV was primarily used for SAV patients, while BEV was mainly applied to TAV patients, creating inherent differences that complicate direct comparisons. Definite transcatheter valve selection was per the operator’s discretion and may introduce selection bias. There was no independent core laboratory for the evaluation of invasive and TTE assessments; however, all data were generated by experienced imagers and hemodynamic technicians. Accordingly, our findings should be considered hypothesis-generating and warrant confirmation in larger studies.

## Conclusion

Mean gradients after TAV in a failing bioprosthesis are similar for BEV and SEV when measured invasively, but higher with BEV than SEV by pre-discharge and 12-month echocardiography. There is a larger discordance between invasive and echocardiography-derived mean gradients with BEV than SEV. Further research is required to understand the clinical relevance of these findings.

## Supplementary Information

Table S1: Valve size and modality measurement discordance: difference between pre-discharge echo mean gradient vs. post-procedural invasive mean gradient (mmHg) across various valve size groups. Table S2: Clinical outcomes

## Data Availability

The data that support the findings of this study are available on request from the corresponding author. The data are not publicly available due to privacy or ethical restrictions.
