# Supplementary material for: Invasive and echocardiographic gradients of self- and balloon-expandable valves in failing aortic bioprostheses
Source: Neth Heart J. 2026 Aug 18;34(9):312–22. doi: 10.1007/s12471-026-02061-7 (PMC13504006; doi:10.1007/s12471-026-02061-7)
Supplement: Supplementary file 1 [file 12471_2026_2061_MOESM1_ESM.docx]

**Supplement Invasive and Echocardiographic Gradients of Self- and Balloon-Expandable Valves in Failing Aortic Bioprostheses**

**Supplementary Table 1.** Valve size and modality measurement discordance: difference between pre-discharge echo mean gradient vs. post-procedural invasive mean gradient (mmHg) across various valve size groups (SEV vs BEV)

| Valve size | **SEV  (reference  = 4.4)** | **P**  **Value*** | **BEV  (reference = 12.8)** | **P  value*** | **P value  (SEV vs. BEV**) |
| --- | --- | --- | --- | --- | --- |
| Very small (XS) (Sapien3 20 mm or  Evolut 23mm) | 14.0 (5.0-*NA**)* | Reference | 12.0 (12.0-12.0) | Reference | 1.000 |
| Small (S) (Sapien3 23 mm or  Evolut 26mm) | 2.0 (-2.0-6.5) | **0.025** | 13.5 (10.8-16.3) | 0.750 | **0.001** |
| Medium (M)  (Sapien3 26 mm or  Evolut 29mm) | 3.0 (1.3-6.5) | **0.049** | 5.7 (2.8-11.4) | 1.000 | 0.129 |
| Large (L) (Sapien3 29 mm or  Evolut 31/34mm) | *NA*** | *N/A*** | 14.5 (12.0-*NA**)* | 0.667 | *N/A*** |
| Values are median (25^th^-75^th^ percentile). *For comparisons between valve size categories, smallest valve size (XS) is used as reference category. Other comparisons are as follows for SEV (S vs. M: p=0.648; S vs. L: p= *NA***; M vs. L: p= *NA***) and for BEV (S vs. M: p=0.106; S vs. L: p=0.889; M vs. L: p=0.381)  SEV (n): S (4), S-M (17), M (15), L(0), BEV (n): S (1), S-M (9), M (6), L(4)  **Insufficient data for comparison or calculation | | | | | |

**Supplementary Table 2**. Clinical outcomes

|  | SEV (n = 36) | BEV (n = 20) |
| --- | --- | --- |
| 30 days* |  |  |
| All-cause mortality | 1 (3%) | 0 (0%) |
| Cardiovascular mortality | 1 (3%) | 0 (0%) |
| Myocardial infarction | 0 (0%) | 0 (0%) |
| Bleeding | 0 (0%) | 0 (0%) |
| Vascular complication | 0 (0%) | 1 (5%) |
| Pacemaker implantation | 0 (0%) | 0 (0%) |
| Rehospitalisation | 0 (0%) | 1 (5%) |
| Rehospitalisation for heart failure | 0 (0%) | 0 (0%) |
| Stroke | 0 (0%) | 0 (0%) |
|  |  |  |
| NYHA Class |  | *p-value = 0.629* |
| - NYHA 1 | 24 (67%) | 9 (45%) |
| - NYHA 2 | 6 (17%) | 4 (20%) |
| - NYHA 3 | 1 (3%) | 1 (5%) |
| - NYHA 4 | 0 (0%) | 0 (0%) |
| Change in NYHA Class | -2 (-2 - -1) | -1 (-2 - -1) |
|  |  |  |
| 12 months* |  |  |
| All-cause mortality | 2 (6%) | 1 (5%) |
| Cardiovascular mortality | 1 (3%) | 0 (0%) |
| Myocardial infarction | 0 (0%) | 0 (0%) |
| Bleeding | 0 (0%) | 0 (0%) |
| Vascular complication | 0 (0%) | 1 (5%) |
| Pacemaker implantation | 0 (0%) | 0 (0%) |
| Rehospitalisation | 2 (6%) | 2 (10%) |
| Rehospitalisation for heart failure | 0 (0%) | 0 (0%) |
| Stroke | 1 (3%) | 0 (0%) |
|  |  |  |
| NYHA Class |  | *p-value = 0.886* |
| - NYHA 1 | 14 | 7 |
| - NYHA 2 | 11 | 4 |
| - NYHA 3 | 4 | 2 |
| - NYHA 4 | 1 | 0 |
| Change in NYHA Class | -2 (-2 - -1) | -1 (-2-0) |
| Values are median (25^th^-75^th^ percentile) or n (%) BEV = balloon-expandable valve; SEV = self-expanding valve;  *Clinical outcome data available of n = 31 SEV and n = 14 BEV at 30 days, n = 30 SEV and n= 13 BEV at 12 months (otherwise lost to follow-up/death) | | |
